# An approach for dynamical network reconstruction of simple network motifs

**DOI:** 10.1186/1752-0509-7-S6-S4

**Published:** 2013-12-13

**Authors:** Masahiko Nakatsui, Michihiro Araki, Akihiko Kondo

**Affiliations:** 1 Department of Chemical Science and Engineering, Graduate School of Engineering, Kobe University, 1-1 Rokkodai-cho, Nada-ku, Kobe 657-8501, Japan; 2 Organization of Science and Technology, Kobe University, 1-1 Rokkodai-cho, Nada-ku, Kobe 657-8501, Japan

## Abstract

**Background:**

One of the most important projects in the post-genome-era is the systemic identification of biological network. The almost of studies for network identification focused on the improvement of computational efficiency in large-scale network inference of complex system with cyclic relations and few attempted have been done for answering practical problem occurred in real biological systems. In this study, we focused to evaluate inferring performance of our previously proposed method for inferring biological network on simple network motifs.

**Results:**

We evaluated the network inferring accuracy and efficiency of our previously proposed network inferring algorithm, by using 6 kinds of repeated appearance of highly significant network motifs in the regulatory network of *E. coli *proposed by Shen-Orr *et al *and Herrgård *et al*, and 2 kinds of network motif in *S. cerevisiae *proposed by Lee *et. al*. As a result, our method could reconstruct about 40% of interactions in network motif from time-series data set. Moreover the introduction of time-series data of one-factor disrupted model could remarkably improved the performance of network inference.

**Conclusions:**

The results of network inference examination of E. coli network motif shows that our network inferring algorithm was able to apply to typical topology of biological network. A continuous examination of inferring well established network motif in biology would strengthen the applicability of our algorithm to the realistic biological network.

## Background

The investigation of network dynamics in biology is a major issue in systems and synthetic biology. Recent advances in high-throughput technologies for comprehensive observation of cells produce a lot of data for analyzing dynamics of complex system such as gene regulatory networks and metabolic pathways. Time-series with dynamic behavior are one of such data involving enormous amount of information regarding the regulation of biological network *in vivo*. However, as such information is entirely implicit, it requires the development of adequate analytic and computational methods to reconstruct biological systems. The key in developing such computational methods is to build a reliable mathematical model for analyzing biological networks, and to explore parameter values in the model within vast searching space. Tominaga *et al*. and Maki *et al*. have developed a novel method [1,2] inferring conceptual biological networks by the combination of a dynamical network model called S-system [3] with a traditional parameter estimation based on simple genetic algorithms [4,5]. The S-system is based on an ordinary differential equation, in which the temporal (time-dependent) dynamic process of system components are characterized by power-law formalism. The S-system is suitable for conceptual modeling and describing complex systems with a loop or a cyclic interaction because the dynamic behavior of the network can be easily obtained by numerical integration and customized [6]. The values of interrelated coefficients in the formalism are directly or indirectly related to the regulation mechanism in the network model. The inferred network structure from the inference of parameters provides one of the best candidates for the biological network structure. However, S-system requires a large number of parameters that must be estimated to identify dynamical biological networks; the number of estimated parameters is 2*n*(*n *+ 1) (where *n *is the number of system components).

We previously proposed efficient procedures for inferring biological network based on experimentally observed time-series data of mRNA or metabolites [7-10] using S-system and real-coded genetic algorithms (RCGAs) [11] with a combination of uni-modal normal distribution crossover(UNDX) [12] and minimal generation gap(MGG) [13]. Other groups have also developed several methods to optimize parameters using S-system [14-19], Beside of S-system modeling, a lot of network reconstruction algorithms from time-series have been developed [20-27]. However, most of the works focused on the improvement of computational efficiency in large-scale network inference of complex systems with cyclic relations and few attempts have been done for answering practical problems occurred in real biological systems. Herrgård *et. al*., Shen-Orr *et. al*., and Lee *et. al*. proposed that the gene regulatory network in *Escherichia coli *or *Saccharomyces cerevisiae *identified by experimental studies is composed of the limited number of network motif; each motif has simple form of relationships between transcription factors and genes [28,29]. Little attention has been paid to evaluate the performance of network inference for such simple network motifs with dynamical modeling, S-system. In this paper, in order to evaluate the inferring performance of our previously proposed network inferring algorithms, we applied our algorithm to 8 kinds of simple form of network motifs proposed by Shen-Orr *et. al*. [29], Herrgård *et. al*. [28], and Lee *et. al*. [30] Shen-Orr *et. al*. and Herrgård *et. al*. suggested repeated appearances of highly significant motifs. Lee *et. al *suggested network motifs based on genome-wide location data.

## Results and discussion

### Results of network identification

**Figure 1:**
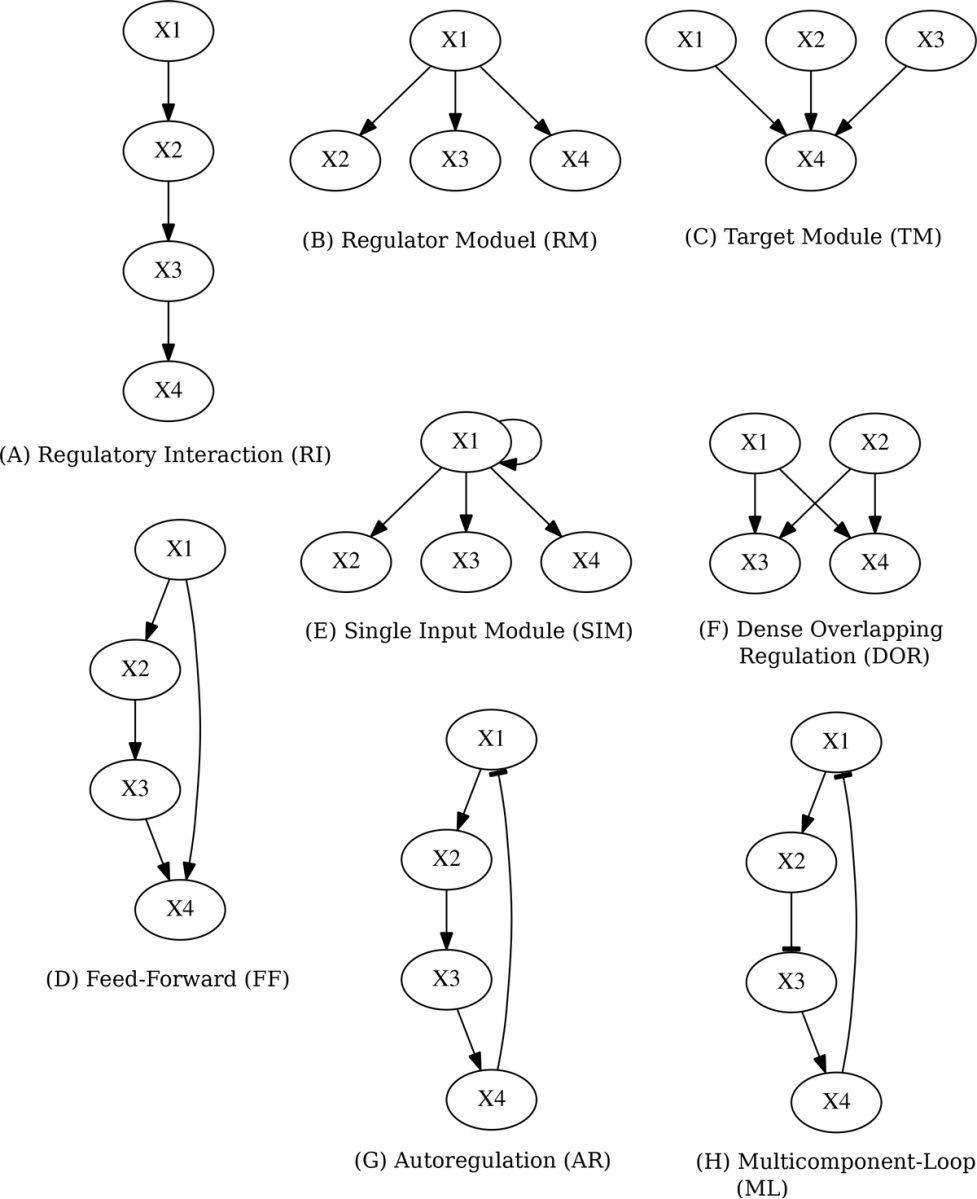
**Network motifs**. The 8 kinds of network motifs. The nodes shown as *X*_1 _to *X*_4 _represent regulator or regulon. RI, RM, and TM were modified by network motifs proposed by Herrgård *et al*. The structures of RI, RM, and TM were constructed according to the same network motif proposed by Herrgård *et al*. The structures of RI, RM, and TM were constructed according to the same concept of network motif proposed by Shen-Orr *et al*. The structures of AR and ML were constructed according to the same concept of network motif proposed by Lee *al*. (A) Regulatory Interaction (RI): a factor *X*_1 _regulates *X*_2_, *X*_2 _regulates *X*_3_, and *X*_3 _regulates *X*_4_. (B) Regulator Module (RM): a factor *X*_1 _regulates a set of regulons shown as *X*_2 _to *X*_4_. (C) Target Module (TM): a regulon *X*_4 _is regulated by other factors shown as *X*_1 _to *X*_4_. (D) Feed-Forward (FF): a regulator *X*_1 _regulates *X*_2_, and both jointly regulates *X*_4_. (E) Single Input Module (SIM): same as RM, a factor *X*1 regulates a set of regulons shown as *X*_2 _to *X*_4_, and also regulates *X*_1 _itself. (F) Dense Overlapping Regulation(AR): regulons *X*_3 _and *X*_4 _were regulated by regulator *X*_1 _and *X*_2_, respectively. (G) Autoregulation: *X*_1 _regulates *X*_2_, *X*_2 _regulates *X*_3_, *X*_3 _regulates *X*_4_, and *X*_4 _inhibitory regulates *X*_1_. (F) Multicomponent-Loop (ML): *X*_1 _regulates *X*_2_, *X*_2 _inhibitory regulates *X*_3_, *X*_3 _regulates *X*_4_, and *X*_4 _inhibitory regulates *X*_1_.

**Figure 2:**
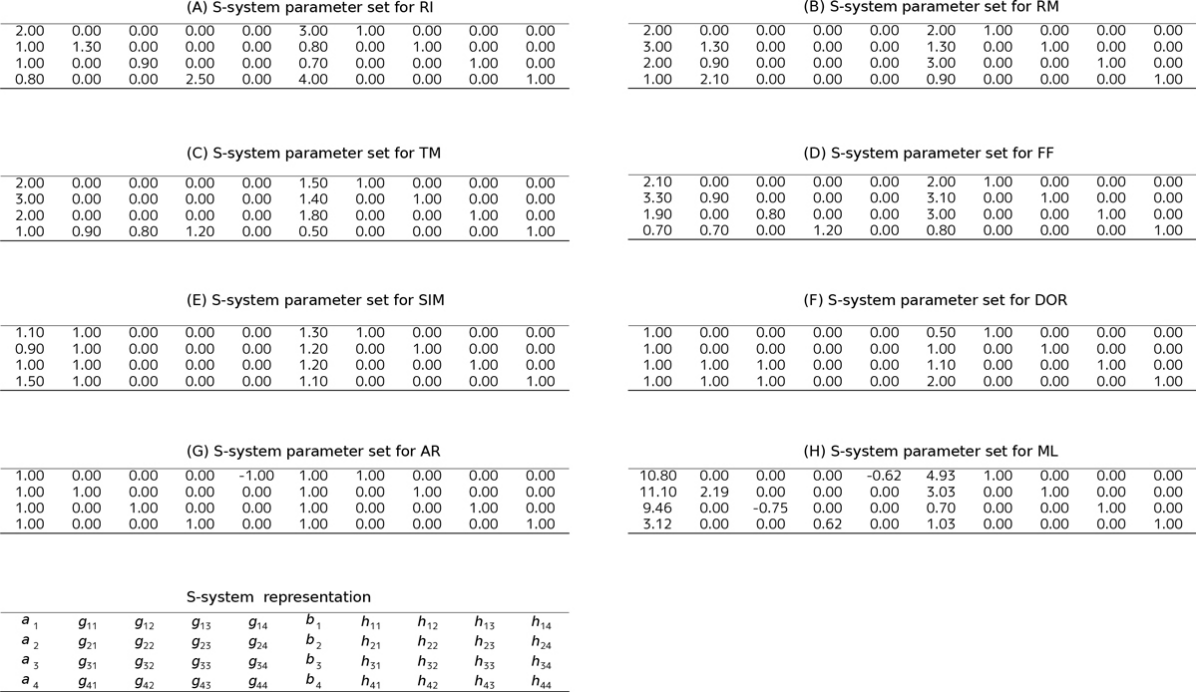
**S-system representations of 8 kinds of network motif**. S-system parameter values for calculating artificial generated time-series (shown in Figure 3). (A) The S-system representation of RI. (B) The S-system representation of RM. (C) The S-system representation of TM. (D) The S-system representation of FF. (E) The S-system representation of SIM. (F) The S-system representation of DOR. (G) The S-system representation of AR. (F) The S-system representation of FF.

**Figure 3:**
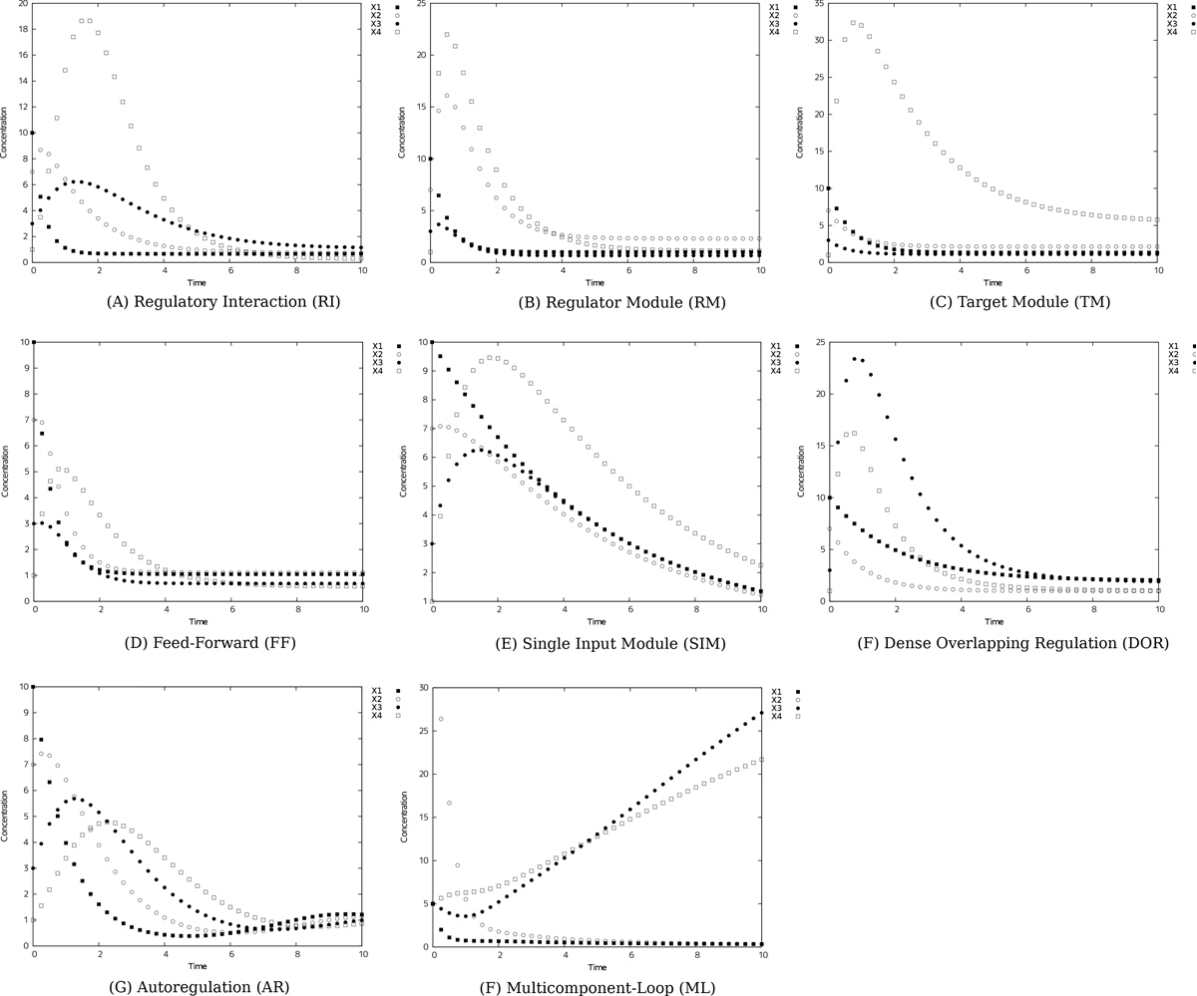
**Reference time-series**. The artificial reference time-series simulated based on 8 kinds of network motif in Figure 1. We prepared these time-series under following conditions: the number of sampling point is 40, initial value of *X*_1 _is 10.0, that of *X*_2 _is 7.0, *X*_3 _is 3.0, and *X*_4 _is 1.0 for RI, RM, TM, FF, SIM, and DOR. For ML, initial values are *X*_1 _= *X*_2 _= *X*_3 _= *X*_4 _= 5.0. We set *h_ii_*, which represents the interrelated coefficient for self degradation, at 1.0, and set other *h_ij _*at 0. (A) The reference time-series of RI. (B) The reference time-series of RM. (C) The reference time-series of TM. (D) The reference time-series of FF. (E) The reference time-series of SIM. (F) The reference time-series of DOR.

**Figure 4:**
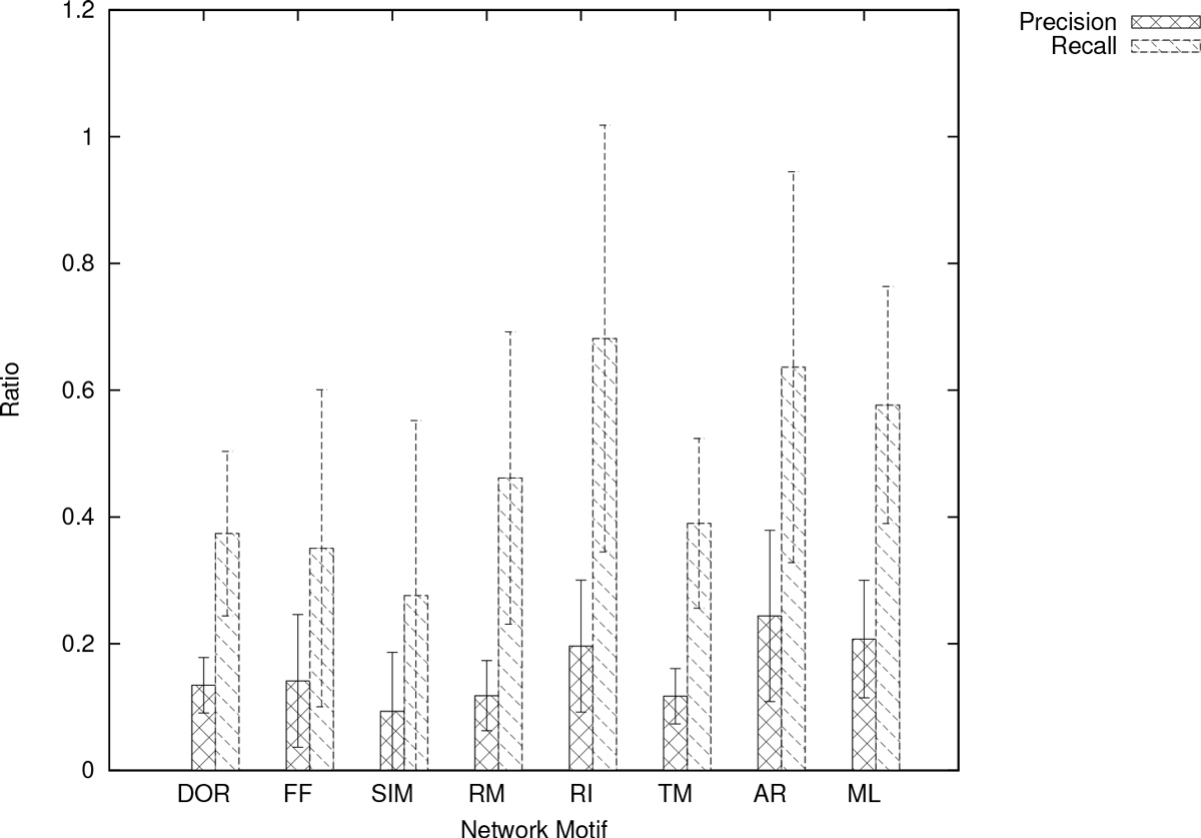
**Network Inferring Performance: Precision, Recall, and F-measure**. The resulting accuracy and efficiency for each network motif.

**Figure 5:**
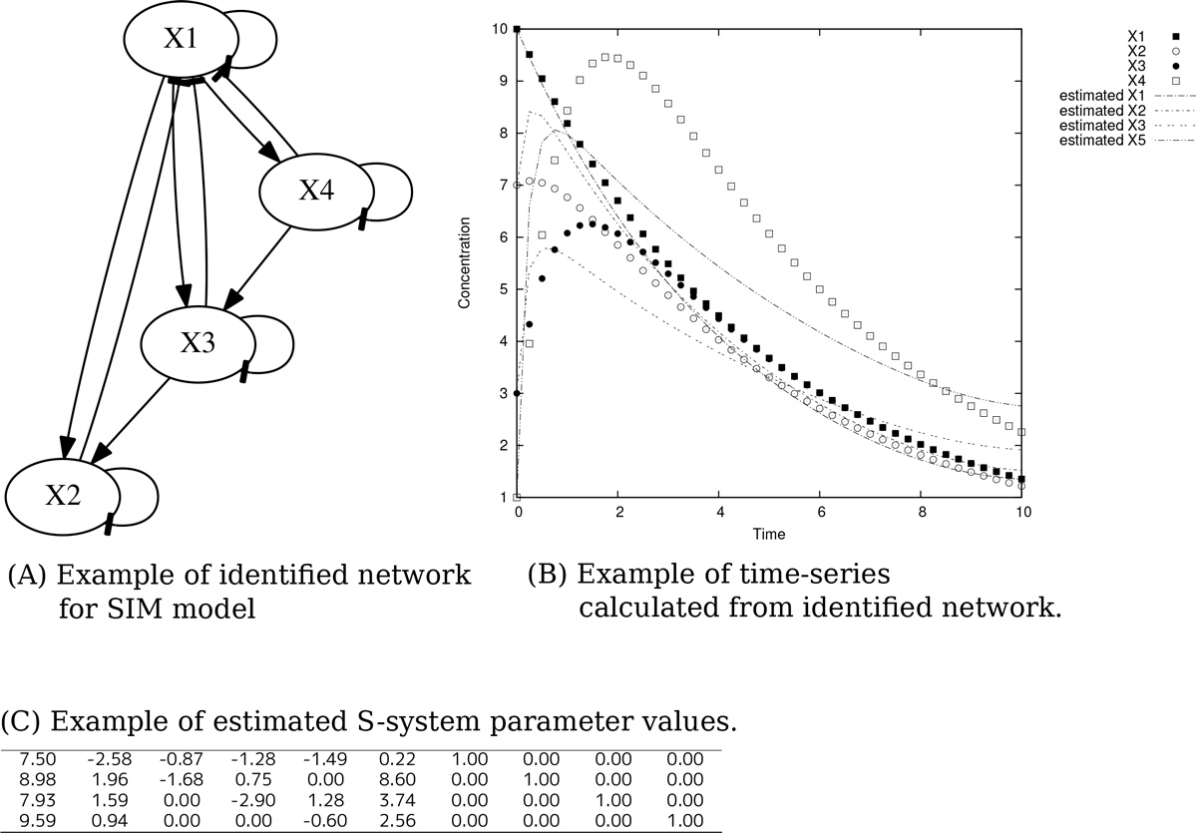
**Example of inferred network**. (A) Example of inferred network based on time-series of SIM (shown in Figure 2 (E)). (B) Comparison of reference time-series and calculated time-series by using inferred network (A). (C) Example of estimated S-system parameter set based on time-series of SIM (shown in Figure 2 (E)).

We inferred network candidates 100 times for each network motif, based on artificial generated time-series data sets (see Figure 1, 2 and 3). After obtaining 100 network candidates, we calculated precision and recall (see Figure 4) to evaluate the accuracy and efficiency of our algorithm from network structural (topological) point of view. In the case of network inferences for DOR(Dense overlapping regulation), FF(Feed-forward), RM(Regulator Module) and TM(Target Module) networks, the value of recall becomes around 0.4, which indicates about 40% of interactions in the network model are properly reconstructed by our algorithm. The better case were for RI(Regulatory Interaction), AR (Autoregulation), and ML(Multicomponent-Loop) network, and our algorithm could reconstruct around 60% of interactions in the network motif. We also calculated F-measure to evaluate balance of accuracy and efficiency of network identification. Also in F-measure, network identification for RI, AR, and ML network represents better estimation results compared with DOR, FF, RM, SIM and TM. On the other hand, the low values of precision were observed in the all cases, indicating that network candidates inferred from our algorithms. The performance of inferring accuracy (precision) was relatively low, namely, the inferred network candidates contain many false-positive interactions. Figure 5(A) shows the best case of identified network topology for SIM (precision : 0.33, recall : 1.0). Figure 5(A) contains all regulatory interactions in network motif for SIM (shown in Figure 1(E)). However, this contains many false-positive interactions, such as self-degradation for synthetic process, and inhibitory regulation from X2, X3, and X4 to X1.

However, the lower values of precision were often observed in our previous works applied to other types of networks, so that we have already developed a method to remove the false-positive interactions inferred by parallel computing [7,8]. Even though we can apply our previously proposed method to improve the precision values, our aim here is to see how both precision and recall values can be improved by altering the information content of time-series data.

**Figure 6:**
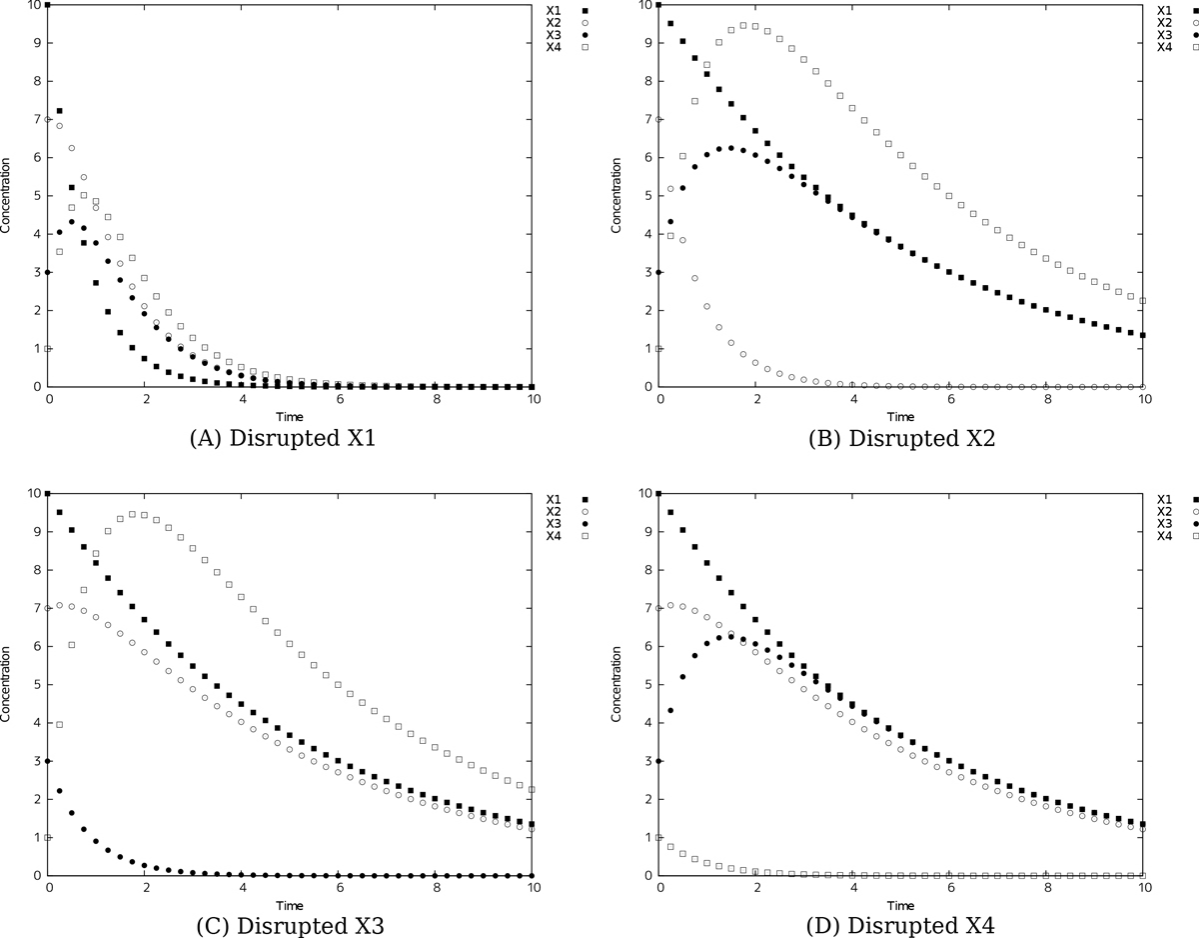
**Reference time-series of SIM for one-factor disrupted strain**. The artificial reference time-series simulated based on same S-system parameter set shown in Figure 2 except the rate constant for the synthetic process of disrupted factor *i *(*α_i_*). We calculated time-series for disrupted strain with *α_i _*set to 0.0.

**Figure 7:**
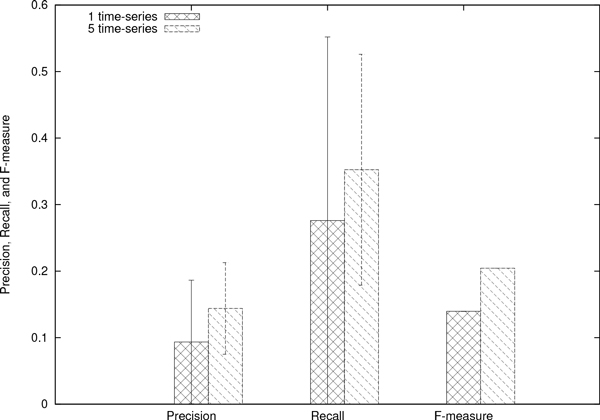
**Comparison of inferring performance with single and 5 time-series**.

We thus focus on the inferred network candidates for SIM since the performance of accuracy and efficiency (Figure 4(E)) was very low. There is a possibility that the imbalance between huge degree of freedom in S-system network modeling and information amount in reference time-series data yields such low performance of accuracy and efficiency. In other words, the information content of the single reference time-series data (shown in Figure 3(E)) is not enough to identify network candidates. To overcome this situation, we tried to infer network candidates by testing another kind of time-series data, more strictly, one-factor disrupted model. We prepared time-series data for one-factor disrupted model as shown in Figure 6. The S-system parameters for reconstructing networks are same as Figure 2 and 3 except the rate constant for the synthetic process of disrupted factor. We prepared time-series data for the one-factor disrupted model with the rate constant for the synthetic process of disrupted factor *i*(*α_i_*) set to 0.0. We inferred 8 network candidates from 5 time-series data including wild-type (see Figure 3(E)) and one-factor disrupted strain. The comparison between single and 5 time-series in inferring accuracy and efficiency is shown in Figure 7. The result shows that the performance is remarkably improved compared with the case in single time-series. We applied the same data to other motifs (data not shown) and found that the introduction of time-series data using one-factor disrupted model can improve the performance of our algorithm.

## Conclusions

We applied our previously proposed algorithm to the network motifs proposed by Herrgård and Shen-Orr. As a result, the efficiency (recall) of our method exhibited relatively high in most of network motifs. In particular, in the Regulatory Interactions (RI) model, we reconstructed about 68% of interactions in the model. Interestingly, the performance of network inference for complex regulatory network including cyclic interactions (AR and ML) was better than that for simple network analyzed in this study. It is likely that the abundant information related to dynamic behavior contained in time-series data for complex regulatory network constrains the degree of freedom S-system modeling, for this reason, the false-positive or false-negative interactions for complex network are reduced.

In order to examine how to improve both the accuracy and efficiency, we attempted to infer the network candidates based on 5 time-series data including time-series for one-factor disrupted model. In this situation, the performance of inferring accuracy and efficiency remarkably increased. This result suggests that the inferring performance can be improved by adding other kinds of time-series data.

Note that the present performance is examined by a set of data generated from arbitrary given parameter values. We should test the performance of our method for various structures of networks with different parameters as well as for observed data. From practical point of view, there have been various kinds of data accumulated under different experimental conditions. The differential information content of such data is expected to further improve the performance of our method. A continuous examination of inferring well-established network motifs in biology would strengthen the applicability of our algorithm to the realistic biological network including gene regulatory networks or metabolic pathways.

## Methods

### Material

In order to evaluate the applicability of our inferring algorithm, we prepared 8 kinds of artificial network models, Regulatory Interaction (RI), Regulator Module (RM), Target Module (TM), Feed-Forward (FF), Single Input Module (SIM), Dense Overlapping Regulation (DOR), Autoregulation (AR), and Multicomponent-Loop (ML). Each network model contains a significant network motif in the regulatory network of *Escherichia coli *proposed by Shen-Orr *et. al*. and Herrgård *et al *[28,29], and that of *Saccharomyces cerevisiae *proposed by Lee *et. al *[30]. We modified the 8 network motifs to network models consisting of 4 nodes (*X*_1_, *X*_2_, *X*_3_, and *X*_4_) without a loss of each network topology. Figure 1 shows each network structure analyzed in this paper.

Subsequently, we prepared artificial time-series data containing 40 sampling points for each network motif by the numerical integration [6]. The reference time-series data of 8 network models are shown in Figure 2.

### S-system formalism

S-system is a suitable formalism for dealing with gene expression network or conceptual metabolic pathway structures. It can sufficiently represent the structure of organizationally complex system, to capture the essence of experimentally observed response:

(1) $$\frac{dX_{i}}{dt}=\alpha_{i}\overset{n}{\underset{j=1}{\prod }}X_{j}^{g_{ij}}-\beta_{i}\;\overset{n}{\underset{j=1}{\prod }}X_{j}^{h_{ij}}\;\left(i=1,2,...,n\right)$$

where *n *is the number of system components (genes or metabolites) in the investigating network, *X_i _*is the experimentally observed response (gene expression level for gene expression network, or concentration of metabolites for metabolic pathway's investigation), *α_i _*and *β_i _*are apparent positive rate constant, and *g_ij _*and *h_ij _*are interrelated coefficients between *X_i_*s.

The first term on the right-hand side of Eq. (1) corresponds to the synthetic process of *X_i_*, and the second term expresses the degradation process of *X_i_*. The value of *g_ij_*(*h_ij_*) express the interactive effects of *X_j _*to the synthetic process (degradation process) of *X_i_*. The value of *g_ij_*(*h_ij_*) also determine the structure of the interactions between *X_i _*and *X_j_*. When the value of *g_ij_*(*h_ij_*) is positive, *X_j _*induces a synthetic process (degradation process) of *X_i_*. On the other hand, when *g_ij_*(*h_ij_*) is negative, *X_j _*suppresses the synthetic process (degradation process) of *X_i_*. When the value of *g_ij_*(*h_ij_*) is zero, then there are no effects of *X_j _*on the synthetic (degradation) process of *X_j_*.

The biological network can be inferred by estimating *α_i_*, *β_i_*, and *h_ij _*in the S-system formula. A representation of S-system parameters to be estimated is shown in Figure 1.

### Real-coded genetic algorithms

The S-system is a formalism of ordinary non-linear differential equations, and thus the system can easily be solved numerically by using numerical integration algorithm customized specifically for this formalism [6]. However, when an adequate time-course of relevant state variable is given, a set of parameter values *α_i_*, *β_i_*, *g_ij_*, and *h_ij_*, in many cases, will not be uniquely determined, because it is highly possible that the other set of parameter values will also show a similar time-course. Therefore, even if one set of parameter values that could explain the observed time-course is obtained, this set is still one of the best candidates that explain the observed time-courses. Our strategy is to explore and exploit these candidates within the immense huge searching space of parameter values.

In this problem, each set of parameter values to be estimated is evaluated by using following procedure: Suppose that $X_{d,i,t}^{\text{CAL}}$ is the value of the numerically integrated time-course at time *t *of state variable *X_i _*in the *d*-th data-set, and $X_{d,i,t}^{\text{EXP}}$ represents the experimentally observed time-course at time *t *of *X_i _*in the *d*-th data-set. Sum up the square values of relative error between $X_{d,i,t}^{\text{CAL}}$ and $X_{d,i,t}^{\text{EXP}}$ to get the total relative error *E*;

(2) $$E\;=\;\overset{D}{\underset{d=1}{\sum }}{\overset{N}{\underset{i=1}{\sum }}\overset{T}{\underset{t=1}{\sum }}\left(\frac{X_{d,i,t}^{\exp}-X_{d,i,t}^{\text{cal}}}{X_{d,i,t}^{\exp}}\right)}^{2}$$

where *D *is the total number of data-sets that experimentally observed under the different kind of experimental conditions such as disruption of genes or inhibition of kinase activities, *N *is the total number of experimentally observable state variables and *T *is the total number of sampling points over time in one experimental conditions. The computational task is to find out a set of parameter values that minimizes the objective function *E*. We have developed the efficient computational technique based on real-coded genetic algorithms (RCGAs) as a nonlinear numerical optimization method which is much less likely to be stranded in local minima. This technique is based on the combination of the operator called *uni-modal normal distribution crossover *(UNDX) [12] with the alternation of generation model called *minimal generation gap *(MGG) model [13]. Furthermore, in order to find the skeletal structure (small-size system) of the S-system formalism that explain the experimentally observed response, some of the parameters (*g_ij _*and *h_ij_*), absolute values of which are less than a given threshold value are to be removed (reset to zero) during optimization procedures.

### Evaluation of identified network

We used the precision and the recall for evaluating the inferred biological network candidates. The precision is defined as follows:

(3) $$\text{precision}=\frac{\text{T}{\text{P}}_{\text{all}}}{\text{T}{\text{P}}_{\text{all}}+\text{F}{\text{P}}_{\text{all}}}$$

(4) $$\text{T}{\text{P}}_{\text{all}}=\overset{n}{\underset{i=l}{\sum }}\text{T}{\text{P}}_{i}$$

$$\text{F}{\text{P}}_{\text{all}}=\overset{n}{\underset{i=l}{\sum }}\text{F}{\text{P}}_{i}$$

where TP*_i _*is the number of true-positive interactions in *i*-th network candidate, FP*_i _*is the number of false-positive interactions in *i*-th network candidate, and *n *is the number of inferred network candidates. The value of precision shows the inferring accuracy of biological network candidates. We also used recall, which indicates the inferring efficiency of network candidates as follows:

(5) $$\text{recall}=\frac{\text{T}{\text{P}}_{\text{all}}}{\text{T}{\text{P}}_{\text{all}}+\text{F}{\text{N}}_{\text{all}}}$$

(6) $$\text{T}{\text{P}}_{\text{all}}=\overset{n}{\underset{i=l}{\sum }}\text{T}{\text{P}}_{i}$$

$$\text{F}{\text{N}}_{\text{all}}=\overset{n}{\underset{i=l}{\sum }}\text{F}{\text{N}}_{i}$$

where FN*_i _*is the number of false-negative interactions in *i*-th network candidates. Both precision and recall values are defined between 0.0 to 1.0, and the best value of precision and recall are 1.0.

For evaluating balance using both precision and recall, defined as follows:

(7) $$\text{F - measure}\;\text{ = }\;\frac{2*\text{precision}\;*\;\text{recall}}{\text{precision}\;\text{ + }\;\text{recall}}$$

## Competing interests

The authors declare that they have no competing interests.

## Authors' contributions

The study was designed by MN, MA, and AK. Analysis was carried out by MN. The paper was written by MN and MA. All authors approved of the final manuscript.

## Declarations

Publication of this supplement was supported by the commision for Development of Artificial Gene Synthesis Technology for Creating Innovative Biomaterial from the Ministry of Economy, Trade and Industry (METI), Japan.

This article has been published as part of *BMC Systems Biology *Volume 7 Supplement 6, 2013: Selected articles from the 24th International Conference on Genome Informatics (GIW2013). The full contents of the supplement are available online at http://www.biomedcentral.com/bmcsystbiol/supplements/7/S6.
